# Fast implementation of a visual analogue scale (VAS) in an Emergency Department

**DOI:** 10.1186/1757-7241-20-S2-P21

**Published:** 2012-04-16

**Authors:** Hanne Jørsboe, Bjarne Fogh, Ann Brockdorff

**Affiliations:** 1 Akutafdelingen, Nykøbing Falster Sygehus, Denmark

## Background

Patient satisfaction in an emergency department highly depends on sufficient treatment of pain. Since the VAS-scale is a useful tool for monitoring pain, the purpose of this study was to investigate if it was possible to implement VAS-scoring in acute patients over a short period of time.

## Methods

A prospective interventional study over 12 weeks. Baseline audits were made weekly 3 times on 25 randomly chosen patient cases. The following interventions were introduced to the triage nurses. All acute patients should be asked about pain using VAS-scale, when arriving to the hospital and the value should be documented together with the vital signs. The nurses were educated in pain treatment and had access to a VAS lineal and a pocket card with treatment algorithm. After the intervention, audits were performed every week on 25 patients through the following 9 weeks. Data will be analyzed with SPC.

Study population was all acutely ill patients older than 15 years, who arrived to the emergency department and had a Glasgow Coma Scale higher than 13.

## Results

Baseline data showed that 22% of the patients with surgical problems (31%) were VAS-scored with a mean score higher than 5. Less than 5 % of the orthopaedic (12%) and patients with medical diseases (57%) were VAS-scored. Already after introducing the nurses to use VAS-lineal at triage, a substantial improvement had happened. 85% of all acute patients were monitored with VAS and followed up, if they had prescribed painkillers. The following weeks will show if it is possible to maintain the result.

## Conclusion

These preliminary data show, that it is possible to implement the use of VAS-scale for assessment of pain over a short period of time, using a simple strategy in a group of nurses, who already work systematically with triage.

